# They paid attention to the whole of me in some way, both physically, mentally, and everything in between: a qualitative study of patients’ experiences of interdisciplinary rehabilitation (PREVSAM) in primary care for musculoskeletal disorders

**DOI:** 10.1080/02813432.2024.2447084

**Published:** 2024-12-28

**Authors:** Annika Ekhammar, Sofia Fridén, Maria E. H. Larsson

**Affiliations:** a Unit of Physiotherapy, Department of Health and Rehabilitation, Institute of Neuroscience and Physiology, Sahlgrenska Academy, University of Gothenburg, Gothenburg, Sweden; b Region Västra Götaland, Primary Care Rehabilitation, Gothenburg, Sweden; c Aktiv Fysio, Mölndal, Sweden; d Region Västra Götaland, Research, Education, Development and Innovation, Primary Health Care, Gothenburg, Sweden

**Keywords:** Musculoskeletal pain, physiotherapy, occupational therapy, teamwork, primary care, qualitative research

## Abstract

**Purpose:**

To explore and describe patients’ experiences and perceptions of rehabilitation according to the rehabilitation model ‘Prevention of sickness absence through early identification and rehabilitation of at-risk patients with musculoskeletal pain’ (PREVSAM).

**Method:**

A qualitative study was conducted, with individual semi-structured interviews analysed using qualitative content analysis. Fifteen patients from three primary care rehabilitation clinics in Sweden who had undergone rehabilitation based on the PREVSAM model participated.

**Results:**

Four categories were identified from the participants’ experiences: Gratitude for the holistic view, Challenging but clarifying to create a health plan, Different needs for addressing work-related factors, and Difficulties and negative experiences. From these categories, an overarching theme was conceptualised: *Grateful for being seen for who I am and given the care I need*.

**Conclusion:**

Participants were generally positive towards the PREVSAM model. The addition of occupational therapy and psychological treatment to physiotherapy was seen by many, albeit not all, as enriching the rehabilitation. Collaboration with the workplace was mainly considered ‘good in theory’. The wide variation in the need for support underscore the importance of person-centredness.

## Introduction

Musculoskeletal disorders (MSDs) may have a substantial impact on peoples’ quality of life as pain and physical disability can significantly affect activities of daily living, workability and may interact with anxiety and depression [1,2]. MSDs account for about 20% of all years lived with disability, placing a substantial burden both to the individual, the employers, the healthcare system, and society in general [1,3].

Musculoskeletal disorders are usually managed in primary care and physiotherapists may be the first point-of-contact when assessing MSDs [4]. Screening for risk factors and using a person-centred and biopsychosocial approach to identify the complexity of the patient’s situation is important in MSD care [5]. However, more knowledge is needed on treatment strategies to prevent acute/subacute problems with pain and functional impairment from turning into chronic problems and sickness absence [6,7]. Recommended treatments for MSDs in primary care are exercise therapy and psychosocial interventions [8]. Moreover, the use of risk-based strategies may prompt healthcare professionals to better explore psychosocial factors in patients seeking care for MSDs. However, healthcare professionals have expressed a preference for making treatment decisions based on their clinical judgement rather than patient’s risk group allocation [9].

The model ‘Prevention of sickness absence through early identification and rehabilitation of at-risk patients with musculoskeletal pain’ (PREVSAM) was designed in Sweden, with the aim to identify patients with acute/subacute MSDs at risk for sickness absence [10,11]. The model’s essential components include early identification of psychological risk factors and interdisciplinary teamwork based on a person-centred and biopsychosocial approach. For the last decades, the biopsychosocial model has been considered an important framework for musculoskeletal research and practice [12,13]. The dynamic interactions between physical, psychological, and social risk factors may lead to a vicious cycle of pain, impaired physical function, and mental illness [14]. A person-centred approach has been interpreted as an important aspect of the biopsychosocial model to recognize and address the complexity of symptoms [15]. The person-centred approach used in the PREVSAM model is based on the model established at the Centre for Person-centred Care, GPCC, at the University of Gothenburg [15,16].

In the PREVSAM model, the core team consists of a physiotherapist, an occupational therapist, and the patient [10,11]. A joint health plan is created that includes the patient’s goals and clarification of responsibilities for actions to be taken. For example, the physiotherapist is responsible for tailoring an exercise program and adjust when needed, the occupational therapist is responsible for giving tailored advice on activities, and the patient is responsible to follow the planning and give feedback on how it works. Early access to psychological treatment is possible, and if requested, a psychotherapist is included in the team. After mapping work-related factors, the healthcare professionals can contact the employer if the patient consents. A short description comparing the PREVSAM model and treatment as usual is presented in Table 1.

**Table 1.** The PREVSAM model compared to treatment as usual for musculoskeletal disorders in primary care rehabilitation.

The PREVSAM model	Treatment as usual
Medical history with a person-centred approach	Medical history, may have a person-centred approach
Physical examination	Physical examination
Assessment of attitudes towards responsibility for managing musculoskeletal problems	
Occupational therapy assessment	May occur
Creation of a joint health plan with structured and synchronised interventions	An individual rehabilitation plan may be created
Interdisciplinary teamwork	Uniprofessional treatment, multiprofessional treatment may occur
Optional psychological treatment	
Optional contact with the workplace	

Qualitative data on how the model is experienced and perceived by the patients who receive the intervention may contribute with important perspectives for a possible implementation in ordinary care. This study was designed to fill the knowledge gap on how patients with acute/subacute MSDs experience and perceive being part of a team with different professions, creating a health plan to prevent their problems from becoming long-term, and addressing work-related factors.

## Aim

The study aimed to explore and describe patients’ experiences and perceptions of rehabilitation according to the PREVSAM model.

## Methods

### Study design and setting

The study employed a qualitative design with semi-structured interviews, and is part of the PREVSAM research project [10]. When this study was conducted, the PREVSAM trial was ongoing at two rehabilitation clinics in Region Västra Götaland and at one rehabilitation clinic in Region Värmland. They are Sweden’s second and eighth largest region, respectively.

The consolidated criteria for reporting qualitative research (COREQ) guidelines were followed (Supplementary Appendix I).

### Participants and procedure

When consenting to participate in the PREVSAM trial, patients who were allocated to the intervention and accepted being contacted for an interview were eligible to participate in this study. Twenty-one patients were sent information about the study and fifteen agreed to participate. They chose time and place for the interview, with options for it to be held at their rehabilitation clinic, at the research centre, by a digital platform, or by telephone. All chose to participate by telephone. The interviews started with the participants confirming that they had received written information about the study and gave their verbal consent to be interviewed.

### Data collection

The second author conducted all interviews, using semi-structured open-ended questions. The interview guide was developed by the authors (Supplementary Appendix II), and all interviews had the same opening question. The components of the PREVSAM model were explored; individual assessments from different healthcare professionals, individualised rehabilitation based on interdisciplinary teamwork and a joint health plan with clear divisions of responsibilities. Optional components were early access to psychological treatment, and workplace contact. After the first two interviews, improvements in interview technique were made, i.e. using more follow-up questions to obtain richer data. The semi-structured interview guide remained the same and the interviews were included in the study. The interviews were conducted in May 2023 and lasted between 12.36 and 40.13 min They were recorded with a Dictaphone, pseudonymised, and transcribed verbatim.

### Research team and reflexivity

The research team consists of three female physiotherapists. The first and second authors (AE, SF) have master’s degrees in medical science with a major in physiotherapy. AE is a PhD student in the PREVSAM project and has previous experience of qualitative research. Both have been working clinically for over thirty years and are currently working in primary care rehabilitation clinics. The last author is the principal investigator of the PREVSAM project and a professor with considerable research experience. She is also a manager for research and development in primary care, with previous long clinical experience. None of the authors had any involvement in the participants’ care. All have an interest in developing rehabilitation in primary care.

### Data analysis

The interviews were analysed using qualitative content analysis as described by Graneheim and Lundman [17, 18]. In the first step, the transcribed interviews were carefully read several times by AE and SF to gain an understanding of the whole material. Next, they processed the text line by line. Based on the purpose, the text was divided into meaning units and relevant text was distinguished from irrelevant text. The meaning units were condensed and labelled with a code, i.e. de-contextualisation. In the next step, the re-contextualisation, the codes were sorted, forming subcategories and categories. In this process, similarities and differences of the codes were compared. Finally, the results were discussed with and approved by the last author. Examples of the analysis are presented in Supplementary Appendix III.

## Results

### Participants

The participants consisted of five men and ten women aged between 35 and 64 years. They had sought care for an MSD and had reported psychological risk factors associated with sickness absence on the Örebro musculoskeletal pain screening questionnaire short form (ÖMPSQ-SF) [19]. In the PREVSAM trial, the cut-off was set to 40p or more. The participants were recruited from three different primary care rehabilitation clinics. They mainly lived outside a big city, although some lived in a small town in a more rural area. One participant started the rehabilitation according to the PREVSAM model during 2020, six during 2021, and eight during 2022. All participants were part of a PREVSAM team. Ten participants chose to take advantage of the optional psychological treatment and one of the optional contact with the workplace.

### Identified categories and theme

**Figure 1.**
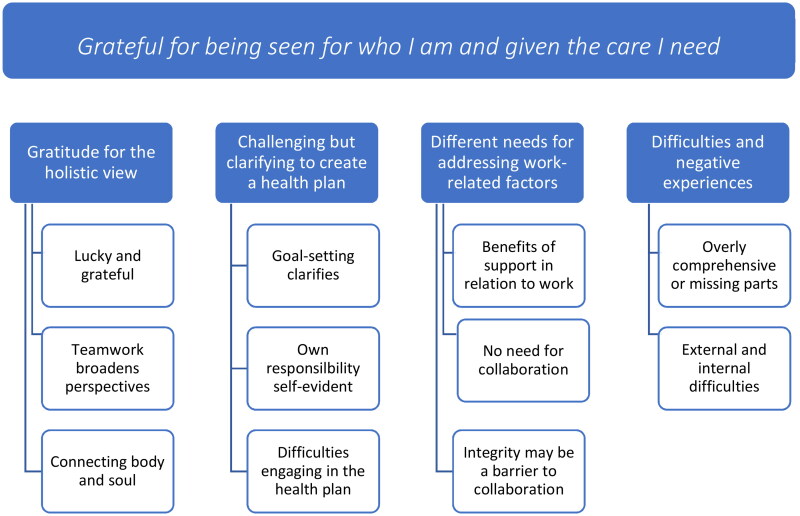
Overarching theme, categories, and sub-categories.

Four categories were identified in the analysis process, each with two or three associated subcategories. From the categories, the overarching theme was conceptualised (Figure 1). The first category relates to participants experiencing the model as having a holistic view and being seen as a person. The second category relates to perceived benefits and challenges with creating a health plan. The third category encompasses perceptions and experiences involving the workplace. The last category highlights perceived difficulties. The overarching theme ‘Grateful for being seen for who I am and given the care I need’ illuminates the meaning of the participants’ experiences and the importance of being seen and treated as a unique person with a need for individually tailored interventions. Being seen as a person gives faith and trust in the rehabilitation; the team members’ being competent, committed; and the individually tailored interventions provided in the health plan seemed to be the basis for the participants experiencing their rehabilitation as meaningful.

### Gratitude for the holistic view

This category underscores how creating a trustful partnership was experienced as a cornerstone. Being part of a team instilled a feeling of being significant and taken seriously, making the participants feel safe and grateful. They described being acknowledged as a person, cared for, encouraged to find ways to feel better, and that ‘both me and my life’ were addressed. This person-centredness provided an appreciated sense of wholeness.

#### Lucky and grateful

The PREVSAM model’s teamwork was considered luxurious, and the participants were grateful for the person-centred approach. Some perceived the rehabilitation quite demanding but worth the effort, while others did not. Participants with previous experiences of poor treatment initially feared they would not receive proper attention. They appreciated that the healthcare professionals were interested, followed up on how they were doing, and provided practical advice.’

‘I think I was lucky to get the help I needed during my journey…//…I agreed to everything to sort of save myself there and I was grateful for all the help and grateful being allowed to be part of this’. (Participant 1)

‘It felt like someone cares, you know…like you mean something. That you’re not just a patient with a number, but you’re a real person’. (Participant 3)

#### Teamwork broadens perspectives

Having a supportive physiotherapist was seen as essential. However, meeting different healthcare professionals was perceived as enriching, contributing to the holistic view. The participants discussed different topics with different team members who were perceived as being experienced, knowledgeable, well prepared, and supportive. They were pleased that the healthcare professionals’ advice was consistent.

‘All the different parts together turned out as good as possible…. although they worked in different places, and you did different things depending on who you met, it felt like they worked together to make me well’. (Participant 8)

The participants who attended the optional psychological treatment received tools to deal with difficulties on a personal level, but also in relation to work or family. They appreciated receiving very helpful and practical advice, such as referral for additional help or guidance on coping with feelings of guilt over being unable to do things as before.

‘So even though I had injured my knee it helped a lot to have someone to talk to. There were other questions and thoughts that came up’. (Participant 8)

#### Connecting body and soul

The person-centred approach made the participants aware of having more than physical ailments, for example stress-related problems. Discussing with the team provided new insights; that psychosocial issues can both contribute to and maintain pain, and that their problems consequently could not be managed with exercise alone.

‘I couldn’t really connect these problems with my shoulder pain, but I understood that later. Because it’s… usually its… complex. It wasn’t just one thing, and it settles in the body in many different ways’. (Participant 9)

### Challenging but clarifying to create a health plan

This category revealed the importance of good collaboration between the participants and the healthcare professionals. Goal-setting clarified what to do and which PREVSAM healthcare professional the participant could benefit from. Creating a health plan was perceived as helpful in adhering to the health plan, and thus to achieve the desired outcomes. However, goal-setting could also be perceived as unnecessary, uncomfortable, or a stressful demand. This perception led to those needing more support feeling guilty and blaming themselves for not being sufficiently independent, which could destroy the partnership and the trust and engagement in the rehabilitation.

#### Goal-setting clarifies

The participants described that goal setting helped them to identify things that mattered. Creating the health plan made it easier to understand what the rehabilitation entailed by providing insights into what needed to be done.

‘If you’re not feeling well mentally, it’s easy to lose yourself, or get lost…When there is a plan it’s easier to stick to that’. (Participant 9)

The healthcare professionals’ support was described as significant during the goal setting, and their knowledge provided security and confidence. Many goals were considered obvious but goal setting and the procedure of dividing goals into subgoals clarified that one cannot reach all the way at once and that small steps in the right direction also count.

‘Sure, it’s pretty obvious, but then I got it written down on a paper, that may have put a little more demand on what to do, it became an ‘easy to do list’ if you put it that way, it’s a form of tool’. (Participant 13)

#### Own responsibility self-evident

The participants experienced being responsible for their own well-being. The healthcare professionals were considered responsible for providing tools and knowledge about proper treatment. The participants themselves must put an effort into following the health plan. Follow-ups and discussions with the team members facilitated taking responsibility for reaching the goals.

‘Responsibility, they didn’t have to tell me about it, I understood that when we talked. I will not back down or give up, I still have my darned exercise bands hanging here. I can see the exercise band every time I pass by, that was my way of taking responsibility, not to hide them away’. (Participant 11)

Even though they saw themselves as responsible, the participants wished for support during tough periods and wanted the healthcare professionals to be responsible for checking on them if they did not come as planned. Ambiguity regarding responsibility was seen as a weakness that affected both continuity and commitment to rehabilitation.

‘Healthcare must ensure that you have tools and opportunities to perform and get the help you need, but it’s above all the responsibility of the individual’. (Participant 12)

#### Difficulties engaging in the health plan

Goal setting could be difficult due to uncertainty about what to expect and what was possible to achieve. Some expressed having a hard time coming up with goals. Some lacked support, some described setting goals that were never followed up. Sometimes the planning led nowhere, and no revision of goals was conducted. However, some experienced that follow-ups at team meetings were tiresome and could be more stressful than helpful. For some, a lack of energy and feeling too bad mentally were barriers to treatment adherence.

‘I can find it a bit stressful with goals and stuff like that. Because I may have difficulty sometimes in expressing myself, or knowing what my goals are or something like that’. (Participant 15)

Some participants perceived that physiotherapy alone would have been enough, they just wanted help to recover from the MSD, and setting other goals was not considered relevant.

‘It happened by itself that I recovered. So, for me I think it kind of didn’t matter’. (Participant 6)

### Different needs for addressing work-related factors

This category highlights that in theory, collaboration between healthcare and employers was perceived as important when needed, but a desire to maintain privacy may lead employees to refrain from such contact. According to some participants, receiving information on work-related factors was valuable. When there was already a good relation between the employee and the employer or when the MSDs did not affect work ability, collaboration between healthcare and the workplace was considered unnecessary.

#### Benefits of support in relation to work

The participants appreciated discussing how their MSDs affected their work ability and receiving advice on how to manage the situation.

‘We discussed my injury in relation to work…//…how I can stand and some other things to make it easier for my back and knees and like, you can sit down sometimes’. (Participant 13)

Discussing employers’ responsibilities was experienced as helpful. Experiences were voiced of not all employers being responsive and interested in helping them during the rehabilitation period. The participants described experiences of employers not willing to offer alternative work tasks, or making necessary adjustments, and they lacked an understanding and flexible attitude. Support from the healthcare professionals was perceived valuable; although seldom requested, a dialogue meeting with the employer was perceived as a reassuring possible backup.

‘I think it’s good that the opportunity exists, that the occupational therapist who is responsible can make contact and do the talk if you have difficulties speaking on your own and need support’. (Participant 10)

However, difficulties were also described. When the collaboration did not work well it was experienced more as a disappointment, due to the healthcare professional lacking a mandate to make work adjustment decisions.

‘The workplace visit felt almost unnecessary. My boss wasn’t there, no…//… I wish it was clearer from the healthcare what the employer can help with, I lacked a commitment to it’. (Participant 14)

#### No need for collaboration

For those with MSDs that directly impacted their work ability, actions were typically already taken by the employer, such as assigning alternative work tasks or providing assistive devices. Many participants described having a good understanding from the employer, and found it easy to solve problems at the workplace without involving the healthcare professionals.

‘Yes, I have a very good boss and she had already ordered special equipment for support… I think I’ve gained a lot of understanding’. (Participant 2)

Participants whose MSD did not interfere with work felt no need to inform the employer and a collaboration between healthcare and their workplace was consequently perceived unnecessary.

‘I did not raise the problems with my employer as work was going well’ (Participant 10)

#### Integrity may be a barrier to collaboration

The relationship with the employer, and the employee’s need for privacy were two important factors that determined whether the participant whose work ability was affected wanted to include the employer during their rehabilitation or not. Some participants did not want the employer to know about their problems, especially if not work-related. This reluctance could be due to a strong desire to manage the situation themselves, fear of not being taken seriously, or a fear of jeopardising their livelihood.

‘I absolutely did not want to be on sick leave. They have wanted to write me off sick, but I have refused, the problem is not my work, I must be able to provide for myself’. (Participant 4)

In retrospect, some participants expressed regret for not having taken advantage of the offer of collaboration between the healthcare and the employer, as it could have led to improved understanding of their difficulty to perform certain tasks.

‘My employer knew nothing…in a way it would have been good if they knew that you might not be able to do heavier work all day…it would have been nice if the employer had known, then maybe I wouldn’t have felt the same pressure or what to say’. (Participant 8)

### Difficulties and negative experiences

This category summarises the participants’ different experiences of their rehabilitation according to the PREVSAM model not working satisfactorily. This experience could be due to their perception of the model being too comprehensive or them not having the time and energy needed to address several factors. Some lacked factors important to them or lacked commitment from the healthcare professionals.

#### Overly comprehensive or missing parts

Some participants only wanted help with physical function and perceived team-based rehabilitation as redundant. However, they were open for others to benefit from it.

‘In many cases it can probably be good to meet several professionals, but for me physiotherapy would have been enough’ (Participant 14)

Some participants expressed that they wished that the general practitioner (GP) should have been involved in the team. Sometimes the healthcare professionals contacted the GP for treatment advice, but the participants expressed missing the GP’s interest in following up on the outcome of their rehabilitation and that they would have appreciated a better collaboration between the GP and the team members.

‘The GP doesn’t follow up at all, that’s bad’ (Participant 1)

The participants would have appreciated being contacted also when they themselves had cancelled; to feel missed and asked for would have been a comfort. When commitment from the healthcare professionals was not forthcoming, those needing more support were afraid not being able to manage the rehabilitation themselves. Some lacked follow-up meetings after completion of the rehabilitation period. They perceived it disappointing that the healthcare professionals did not seem to want to know how things had turned out for them.

‘One might, you know, fall through the cracks like that. You want to get help not to do that. Unfortunately, that can happen. That’s how it is’. (Informant 9)

Despite the PREVSAM model’s biopsychosocial approach, views were raised that not all aspects of being human were addressed, indicating a perceived lack of awareness of spiritual dimensions regardless of beliefs in the PREVSAM model. It was suggested that recognising human beings not only as body and soul but also spirit should be acknowledged in a rehabilitation model aiming to have a holistic view.

‘I missed the spiritual dimension… you see the human being as a body which only has a soul… I therefore think that the spiritual part should also be included, a pastoral care provider as an additional treatment… that part must not be omitted’. (Participant 3)

#### External and internal difficulties

Some participants described that even if the PREVSAM model was relevant for their problems, it was not the right time for them to participate. The bad timing could, for example, be related to the Covid-19 pandemic or to private matters making it difficult for them to focus on the health plan. Feeling too bad mentally was another reason making it difficult to engage in the rehabilitation.

‘I thought it was good, but my mental wellbeing crashed soon after that…the pandemic didn’t make things any better with the huge existing workload, it was just too much. Maybe this was the time when I should have needed the team, but since I backed out myself, it didn’t happen that way’. (Informant 12)

The pain condition itself and other health-related problems took a lot of energy, and some expressed that it was too demanding to be committed to the health plan when lacking energy. Whether it was due to lack of time or energy, having several meetings and appointments made some drop out of their rehabilitation.

‘I think the whole thing fizzled out. But that may also depend a little on me, of course, but I think that the whole thing actually fizzled out… being in pain makes you so tired’. (Participant 14)

## Discussion

The main finding of this study was that the participants generally appreciated rehabilitation according to the PREVSAM model. The meaning of the theme *Grateful for being seen for who I am and given the care I need* is that the respectful partnership made the participants feeling seen as a person, and the holistic view gave them a deeper awareness of interacting biopsychosocial factors. Trust seems to be an important mechanism for successful rehabilitation, and a person-centred approach enables a trustful and respectful partnership.

In the category *Gratitude for the holistic view,* our participants underscored the importance of being listened to, taken seriously, and followed up. When receiving such care, they felt it was luxurious to be part of the rehabilitation. This is in line with the PREVSAM healthcare professionals’ experiences; that it must be almost luxury to have committed healthcare professionals involved in one’s life [20]. The importance of being taken seriously has been highlighted in previous research. It was part of the main theme in a qualitative study by Samsson et al. [21] on patients with MSDs, whose wishes when referred for orthopaedic consultation included being listened to, respected, and receiving information. In a study by Andreasen et al. [22], the element of being taken seriously was essential and reassuring for the participants. When patients feel uncertain about whether they are being taken seriously, the ability to cooperate with the healthcare professionals can be impaired [23]. This cooperation is important, as a good therapeutic alliance can contribute to adherence to the musculoskeletal physiotherapy intervention [24]. Bernhardsson et al. [25] found that trust in the physiotherapist’s competence was important to enhance engagement in treatment, regardless of both preferred treatment methods and preferred role in decision making. The approach of not letting the patient drop out from the rehabilitation without an explanation was considered important according to the PREVSAM healthcare professionals [20]. The importance of making the patients trust them and showing the patients that they as professionals are there for them ‘for better and for worse’ was underscored. The feeling of being taken care of was experienced by most, albeit not all, of our participants.

The person-centred approach in the PREVSAM model was valued by our participants. They expressed appreciation of being recognised as a person, and of the model considering the complexity of health. These findings are consistent with previous literature underscoring the importance of person-centredness. Calner et al. [26] found that patients expect, and desire, to be both seen and affirmed as a person and to be given individually designed treatment with frequent follow-ups. In a systematic review of qualitative studies, Wijma et al. [27] suggested that goal-setting, individualised treatment, education, and communication, delivered by a skilled physiotherapist, should be core components in person-centredness in physiotherapy. This is in line with of most of our participants’ experiences; that healthcare staff were skilled and provided consistent advice and individualised treatment. Creating a health plan was for many, but not all, perceived helpful.

All participants in this study had contacted healthcare due to acute or subacute MSD, and reported psychological risk factors on ÖMPSQ-SF, as this was an inclusion criterion at the start of their rehabilitation. Many participants had sought care during a period in life when they struggled with other problems, such as divorce, illness within their family, or work problems. Their described distressing life events illustrate that such factors impacted the results of their screening. Psychological factors are in previous research shown to affect treatment outcome for patients with MSDs and it is recommended to integrate screening for psychological risk into clinical physiotherapy practice [5,19,28]. However, this group of patients does not routinely have access to psychological treatment in primary care in Sweden. The optional psychological treatment in the PREVSAM model was requested by about half of the participants in the PREVSAM trial; however, not all of them took advantage of the opportunity [29]. Our participants described that this opportunity enriched their treatment. In a study by Nicolas et al. [30] workers scoring increased risk of delayed return to work on ÖMPSQ-SF one to three weeks after injury, were offered psychological treatment and efforts to remove barriers for returning to work. At the two-year follow-up, patients in the intervention group reported more than 50% fewer sickness absence days than controls [30]. This emphasises that screening to early identify individuals who might benefit from psychological treatment and/or workplace contact can facilitate return to work [30] and fits well within the structure of the PREVSAM model.

In the category *Clarifying but challenging to create a health plan,* our participants described that goal-setting clarified what they must do. While the health plan was seen as a useful tool and sub-goals made the rehabilitation more tangible, goal-setting was also perceived as being demanding, difficult or irrelevant. Goal-setting is considered a core component in person-centred physiotherapy [31], giving primacy to the patients and their context, implying a more targeted treatment approach compared with treatment as usual [31,32]. To justify the effort, goals should be meaningful and relevant to the patient [32]. Amundsen et al. [31] found that goals including activity and/or participation factors were associated with better outcomes for patients with MSDs. In our study, some participants described follow-ups being omitted. Both in the PREVSAM intervention and in previous research, fidelity in establishing is higher than following-up health plans [29,33]. A high workload not giving room to contact patients whose appointments have been cancelled, an assumption that it is the patients’ responsibility to book a new appointment, or that the patient does not want to continue with the rehabilitation may explain lack of follow-ups.

Many factors play a role in the complex relationship between MSDs and work ability [34]. Our participants had acute or subacute MSDs when they started their rehabilitation and only few were on sick leave during their rehabilitation. In the category *Different needs for addressing work-related factors,* the participants described different impact on work and different approaches to manage their work situation. It is suggested that an understanding between employers, the employee, and healthcare professionals is fundamental in vocational rehabilitation [34]. Few studies have evaluated prevention of sickness absence for acute/subacute MSDs, and therefore, a knowledge gap remains regarding to whom and when to initiate this collaboration. The offer from the team to contact the employer was rarely used by our participants, which is in line with the overall results from the PREVSAM trial [11] and the healthcare professionals’ experiences [20]. However, according to our participants, it felt safe to know that the option was available. Our results of a great variation in the need for discussing work-related factors are in line with a qualitative study, in which not all participating workers experienced motivational interviewing sessions as useful [35]. Consequently, a person-centred approach to select individuals in need for work-related support is important to avoid providing unnecessary interventions.

For individuals with MSDs and mental health conditions that are already sick-listed, multi-domain interventions are beneficial [36]. Implementing both health-focused interventions, such as exercise and behavioural medicine interventions, improving coordination between workplace and healthcare providers, and making different modifications at work including special equipment as well as modified working hours, is recommended [36]. While experiences varied amongst our participants, health-focused interventions such as exercise were seen as essential, and many valued psychological treatments. Most participants did not perceive a need for workplace contact; they already had a supportive manager who allowed alternative tasks or special equipment, or the MSD did not interfere with their work ability.

In the category *Difficulties and negative experiences,* the sub-category ‘Overly comprehensive or missing parts’ revealed that our participants would have appreciated the GP being more involved, in line with previous research [37]. While most participants praised the healthcare professionals, some felt disappointed. Not feeling strong enough and not being missed when absent were two reasons for disappointment, revealing a resigned attitude amongst those participants. The importance of trust between the patient and healthcare is a keystone when striving for high-quality care. Patients are in an exposed situation and gaining the patients’ trust has been proven crucial to enhance compliance to treatment from both physicians and physiotherapists [25, 38]. On the one hand, previous positive experiences of physiotherapy may instill a sense of trust for physiotherapists as a profession. On the other hand, previous negative experiences may lead to feeling resigned [26].

In line with the PREVSAM model’s claim of being holistic, a wish for optional treatment to address spiritual well-being, regardless of beliefs, was expressed. Spiritual well-being is a human trait that is considered to exist to varying degrees in all individuals [39]. Both social support and spiritual well-being seem to influence coping strategies positively, improve general health, and lead to greater satisfaction after a musculoskeletal injury [40]. Religion and secularity differ between countries and Sweden can be described as one of the worlds most secularised countries. In a survey from 2020 nearly 80% of individuals born in Sweden to Swedish parents reported seeing themselves as secular [41]. Whether extending the PREVSAM model to include optional spiritual guidance would be enriching and possibly more in demand by people with different origins, remains to be investigated.

The participants’ overall life situation likely affected how the model was experienced and according to some participants, physiotherapy alone would have been enough. This is in line with the PREVSAM healthcare professionals’ experiences, highlighting the importance of not only identifying psychological risk factors but also the patients’ needs and resources, before providing team-based interventions [20]. When not all model components were requested, the healthcare professionals expressed different approaches; either they believed that highlighting the patients’ resources was empowering or they believed the mapping was done in vain [20]. These different approaches may have affected the patients’ experiences of rehabilitation according to the PREVSAM model.

### Methodological considerations

The qualitative analysis presents multiple challenges and there are always degrees of interpretation when analysing a text [17,18]. In the analysis, the focus was first on the manifest content, i.e. the visible, obvious components of the text, but then we also discussed the latent content, meaning an interpretation of the underlying meaning of the text. Our pre-understanding of the context may have been an advantage as we could relate to the situations described and to subjects raised. However, it could also be a disadvantage and how to handle our pre-suppositions and assumptions and the inherent risk of confirmation bias were discussed in the research group. Saturation was also discussed amongst the researchers; it refers to the point when no further information regarding the research question is found [42]. However, this point is impossible to determine, and we believe that the participants’ narratives contributed substantially to answer our research question.

A qualitative study’s trustworthiness depends on credibility, dependability, conformability, and transferability of the results, as well as a description of the research team and reflexivity. Regarding credibility, our participants represented three of eight rehabilitation clinics participating in the PREVSAM trial; however, most were from the same clinic which is a limitation. If we had been able to recruit participants from more participating clinics, greater variation in experiences may have been obtained. The reasons why six individuals declined participation are unknown; however, it raises the question of whether they did not value their rehabilitation according to the PREVSAM model enough to consider the interview worth their time. Of the participants, five were men and ten women. This reflects the sex distribution in the PREVSAM trial where 67% were women. The participants all spoke Swedish and came from socio-economically stable areas. However, differences in sex, age, education level, work, and living situation contribute to variation in experiences of rehabilitation. A strength is that the interviewer had no prior connection to the participants and was not involved in the PREVSAM trial. Therefore, the participants likely felt free to express both positive and negative rehabilitation experiences. The interviews were conducted sometime after the rehabilitation period, potentially entailing a risk of recall bias. However, when time has passed the participants may be likely to remember what really mattered to them.

All interviews were made by the same interviewer using the same interview guide, which strengthens dependability. While face-to-face interviews may be superior to telephone interviews, because the lack of visual cues like body language and facial expressions may entail that hidden communication is missed [43], using telephone interviews may have allowed the participants to speak more freely.

Confirmability was achieved by using an established qualitative method [17,18]. Nevertheless, follow-up questions were to some degree influenced by the interviewer’s pre-understanding. How the participants’ descriptions were perceived during the analyses may also have been affected by the researchers pre-understanding [44]. As three researchers participated in the analysis process, the risk of confirming beliefs and producing results based on preconceptions was decreased. Illuminating quotes were used to ensure that the analysis accurately reflects the participants’ responses. Transferability of the results to other settings must take into account both the homogeneity and heterogeneity of the participants.

## Conclusions

The participants experienced the PREVSAM model as a good, holistic rehabilitation model within primary care and valued the person-centred approach. While physiotherapy was perceived as an obvious treatment to address MSDs, the addition of occupational therapy and psychological treatment was seen by many, albeit not all, as enriching the rehabilitation. Although collaboration with the workplace was seldom requested, it was considered ‘good in theory’. The wide variation in the need for support that emerged in the interviews underscore the importance of person-centredness.

## Supplementary Material

Supplemental Material

## Data Availability

The datasets in the current study may be available from the corresponding author on reasonable request.

## Implications for clinical practice and future research

Rehabilitation according to the PREVSAM model may be used in primary care rehabilitation, but an even more flexible approach is needed to identify which patients and healthcare professionals to include in the team and whether goal-setting and creating a health plan is beneficial.

To further investigate possible effects of rehabilitation according to the PREVSAM model, it is desirable to evaluate it in a broader socio-economic context.
