# Supplementary material for: They paid attention to the whole of me in some way, both physically, mentally, and everything in between: a qualitative study of patients’ experiences of interdisciplinary rehabilitation (PREVSAM) in primary care for musculoskeletal disorders
Source: Scand J Prim Health Care. 2024 Dec 28;43(2):380–91. doi: 10.1080/02813432.2024.2447084 (PMC12090256; doi:10.1080/02813432.2024.2447084)
Supplement: Supplemental Material [file IPRI_A_2447084_SM5352.zip › Appendix II Interview guide.docx]

**Appendix II**

**Interview guide ”The patient’s voice in the PREVSAM-study”**

Opening question: When you sought care at the health center because you were in pain, you received treatment according to a new model called the PREVSAM model. Now we would like to know what you think. Can you please tell us how you experienced the interventions, and your thoughts and opinions on the rehabilitation according to the PREVSAM model?

The interviewer will guide the conversation to cover five important areas of the PREVSAM model:

Follow-up questions to clarify and deepen the answers will be used, for example:

-Can you clarify….?

- I would like to know a little more about…

-How have you touched on this at the rehab clinic... (in relation to the five areas above)

-Was there something you missed (and if so, what?)
