# Supplementary material for: They paid attention to the whole of me in some way, both physically, mentally, and everything in between: a qualitative study of patients’ experiences of interdisciplinary rehabilitation (PREVSAM) in primary care for musculoskeletal disorders
Source: Scand J Prim Health Care. 2024 Dec 28;43(2):380–91. doi: 10.1080/02813432.2024.2447084 (PMC12090256; doi:10.1080/02813432.2024.2447084)
Supplement: Supplemental Material [file IPRI_A_2447084_SM5352.zip › Appendix III Examples.docx]

| Theme | **Grateful for being seen for who I am and given the care I need** | | |
| --- | --- | --- | --- |
| Main category | Gratitude for the holistic view | | |
| Sub-categories | Lucky and grateful | Teamwork broadens perspectives | Connecting body and soul |
| Code groups | Surprisingly good  Trust and gratitude | Multi-professional important | Holistic view |
| Meaning Units | *“I was impressed, it was fantastically good, a sense of security”*  *“You get the help you need; they stand up for you”*  *“I am just grateful”*  *“You were taken seriously”* | *“Very nice to get opinions and views from different people”*  *“The psychotherapy was a support, a way to change my mind set, to accept”* | *“I liked the holistic view”*  *“It felt good both physically and mentally”*  *“The treatment covered a lot, both body and soul”* |
| Main category | Challenging but clarifying to create a health plan | | |
| Sub-categories | Goal-setting clarifies | Own responsibility self-evident | Difficulties engaging in the health plan |
| Code groups | Clarifying  Sub-goals helps | Own responsibility obvious  Healthcare professionals | Lack of support and commitment  Lack of energy  Support needed |
| Meaning Units | *“Good to have a plan and get routines”*  *“Made me understand; this will take time”*  *“An ‘easy to do list’, it is a form of tool”* | *“Of course it's my responsibility, they can help me make a plan”*  *“I feel that I have all the responsibility myself”*  *“The health care must ensure that you have tools and opportunities to perform and get the help you need”* | *“The goals were not followed-up”*  *“Gave me nothing, felt like; what are we supposed to do with this?”*  *“It wouldn’t have hurt if someone else had been active and caught me”* |
| Main category | Different needs for addressing work-related factors | | |
| Sub-categories | Benefits of support in relation to work | No need for collaboration | Integrity may be a barrier to collaboration |
| Code groups | Valuable approach | Contact already initiated  Workplace involvement not needed | Privacy important |
| Meaning Units | *“If you get everyone involved, both the care and the employer, it can be a very good overall approach”* | *“I managed the discussion with my boss about my problems”*  *“In my case no dialogue with the employer was needed”* | *“Contact with the employer is about privacy and such, maybe not everyone who wants it”* |
| Main category | Difficulties and negative experiences | |  |
| Sub-categories | Overly comprehensive or missing parts | External and internal difficulties |  |
| Code groups | Too comprehensive program  Missing parts | Wrong time  Feeling too bad |  |
| Meaning Units | *“Meeting a team didn't help me, I didn't suffer from long-term pain”*  *“In my case it would have been enough with the physio”*  *“I lacked follow-ups”*  *“The general practitioner was conspicuous by its absence”*  *“I missed the spiritual dimension”* | *“It was great but then the pandemic broke out and it became too much”*  *“Easy to get lost or lose yourself when you are mentally ill”* |  |

Appendix III. Overarching theme and examples of main categories, sub-categories, code groups and meaning units
